# Genetic variation for sexual dimorphism in developmental traits in *Drosophila melanogaster*

**DOI:** 10.1093/g3journal/jkae010

**Published:** 2024-03-01

**Authors:** Tianyu Li, Rebecca S Zhang, John R True

**Affiliations:** Department of Ecology and Evolution, Stony Brook University, Stony Brook, NY 11794, USA; Department of Biology, Massachusetts Institute of Technology, Cambridge, MA 02139, USA; Department of Ecology and Evolution, Stony Brook University, Stony Brook, NY 11794, USA

**Keywords:** sexual dimorphism, evolvability, *Drosophila*, inbred lines

## Abstract

Sexual dimorphism in traits of insects during the developmental stages could potentially be the direct or indirect result of sex-specific selection provided that genetic variation for sexual dimorphism is present. We investigated genetic variation in sexual dimorphism in a set of *Drosophila melanogaster* inbred lines for 2 traits: egg to adult development time and pupation site preference. We observed considerable genetic variation in sexual dimorphism among lines in both traits. The sexual dimorphic patterns remained relatively consistent across multiple trials, despite both traits being sensitive to environmental conditions. Additionally, we measured 2 sexually dimorphic adult morphological traits in 6 sampled lines and investigated correlations in the sexual dimorphism patterns with the 2 developmental traits. The abundance of genetic variation in sexual dimorphism for *D. melanogaster* developmental traits demonstrated in this study provides evidence for a high degree of evolvability of sex differences in preadult traits in natural populations.

## Introduction

A prevalent characteristic of sexually reproducing animal species is sexual dimorphism. Sexual selection or sex-specific selection is generally responsible for the evolution of sexually dimorphic traits (Shine 1989), which has occurred many times independently across animals (Campbell 1972). This suggests that genetic variation for sexual dimorphism may typically be abundant (Griffin *et al*. 2013; Huang *et al*. 2015), but this has not been thoroughly explored. Model organisms such as *Drosophila* and other insects are ideal candidates for such studies. *Drosophila* have been shown to harbor considerable additive genetic variance in quantitative traits and thus are presumed to have substantial evolvability in these traits (reviewed by Houle 1992).

Quantitative trait evolution can be constrained by genetic correlations (Clark 1987; Futuyma 2010). For example, experimental evolution studies on the eyespot patterns of *Bicyclus anynana* uncovered limitations in response to the antagonistic direction of selection for colors but not for sizes (Beldade *et al*. 2002; Allen *et al*. 2008). Ingleby *et al*. (2014) found that *Drosophila* cuticular hydrocarbons (CHCs) that typically show sexual dimorphic expression are relatively unconstrained from evolving further sexual dimorphism, whereas CHCs that typically do not show sexually dimorphic expression show evidence of constraint against the evolution of sexual dimorphism. Artificial selection for life history traits in *Drosophila* revealed a negative correlation between starvation and cold resistance in females but not in males (Hoffmann *et al*. 2005). Directional selection for extreme trait values or particular trait combinations may be able to decouple correlated traits or may fail due to lack of genetic variation caused by strong genetic correlations between traits. Thus, the evolution of novel sexual dimorphism can be constrained by either a lack of divergent selection between the sexes or the absence of sex-specific genetic variance (i.e. low intersexual genetic correlation; Griffin *et al*. 2013), or both.

Sexually dimorphic adult traits may readily evolve under selection because of genetic variation for traits expressed in the adult stage in which such traits typically function. However, the degree of sexual dimorphism for traits manifesting at earlier stages is generally expected to be lower due to both the lack of selection and sex-specific selection at later stages overriding earlier appearing dimorphic patterns (Badyaev *et al*. 2001; Badyaev 2002). In insects, sexual dimorphism in developmental traits has been observed in several laboratory studies, such as larval body size, larval and pupal development time, and larval feeding strategies (Nunney 1996; Jarošík and Honek 2007; Nunney 2007; Teder 2014; Wormington and Juliano 2014b). In *Aedes* and *Drosophila*, female larvae were found to be larger and foraged longer than male larvae (Wormington and Juliano 2014a; Mathews *et al*. 2017) and adult size followed the same pattern (Huey *et al*. 2006; Wormington and Juliano 2014b). In many insect species, females require more time to develop than males, but *Drosophila* species tend to show the opposite pattern (Nunney 2007; Teder 2014). Sex-specific behaviors such as differential antipredator responses were also observed in *Aedes* larvae during the foraging stage; male larvae were more vigilant toward predator cues than female larvae, which is thought to be an adaptation to differences in energy requirements (Wormington and Juliano 2014a).

In environmentally controlled laboratory experiments, *Drosophila* inbred lines harbor substantial genetic heterogeneity among lines in sexual dimorphism, indicating significant sex-specific genetic variance in the original natural populations (David *et al*. 2005; Mackay and Huang 2018). However, few studies have conducted replicate experiments to account for environmental sources of variation and confirming the repeatability of variation in sexual dimorphism in developmental traits. Sexually dimorphic gene expression is the basis for sexually dimorphic morphology. Huang *et al*. (2015) discovered widespread sex-biased expression in about 90% of *Drosophila* genes using RNA transcriptome sequencing on *Drosophila* Genetic Reference Panel (DGRP) lines, and a substantial proportion (13%) of genes demonstrated significant sex-by-line interaction, i.e. genetic variation in the magnitude of sexual dimorphism between lines. Ayroles *et al*. (2009) quantified genome-wide expressed transcripts in adult *Drosophila* wild-derived inbred lines and found that 40% of the transcripts showed genetic variation in sexual dimorphism. These studies suggest that genetic variation for sexually dimorphic gene expression during development is common in natural populations. We hypothesize that genetic variation in sexual dimorphism in *Drosophila* developmental traits is abundant, which allows natural populations to potentially respond rapidly to sex-specific selection.

This study investigates genetic variation for sexual dimorphism in egg to adult development time and pupation site preference among *Drosophila *melanogaster** inbred lines. In *D. melanogaster* and other *Drosophila* species, females generally emerge slightly earlier than males, on average (Pitnick *et al*. 1995; Bharathi *et al*. 2004; Ashburner *et al*. 2005; Huey *et al*. 2006; Nunney 2007). Nunney (2007) proposed that early eclosion of female *Drosophila* confers a fitness advantage via increased egg provisions prior to sexual maturity. On the other hand, male *Drosophila* may reduce the mortality risk of adulthood in nature before mating by delaying eclosion and reaching reproductive maturity shortly after eclosion (Pitnick *et al*. 1995). According to this hypothesis, there should be heritable phenotypic variation and thus genetic variation in sexual bimaturism in natural populations, which could be captured as differences among inbred lines.

Another potentially sexually dimorphic trait that manifests during development is pupation site preference. A suitable pupation site is required for *D. melanogaster* adults to successfully eclose in the natural environment (Bauer and Sokolowski 1988). By the end of the third instar stage, 4- or 5-day-old *D. melanogaster* larvae cease foraging and begin a 12- to 24-h “wandering” behavior in search of a pupation site, which is typically a relatively dry area (Sokolowski *et al*. 1984). Pupation sites represent a variety of microhabitats that differ in terms of abiotic factors such as texture or substrate type, moisture content, temperature, and light intensity (Manning and Markow 1981; Casares and Carracedo 1987; Medina-Muñoz and Godoy-Herrera 2004; Ramniwas and Kumar 2019). Any of these variables may have an effect on the pupation site selection process. Along with abiotic factors, biotic factors such as species, sex, maternal effects, aggregation (larval density), and CHCs left by adults may influence pupation site preference (Sokolowski *et al*. 1984; Mueller and Sweet 1986; Bauer and Sokolowski 1988; Medina-Muñoz and Godoy-Herrera 2004).

The preference for pupation site is a selectable (and heritable) trait in laboratory populations of *Drosophila*. Ramniwas and Kumar (2019) successfully selected strains of *Drosophila jambulina* that pupate virtually exclusively on cotton or food after 30 generations. They discovered that reciprocal F2 offspring of crosses between differentially selected lines had a Mendelian segregation ratio, suggesting that variation at a single locus was primarily responsible for the selection responses. They also discovered that pupation site preference was highly temperature-dependent; the segregation ratio in F2 shifted from ∼1:3 to 3:1 at 21°C vs 30°C. Bauer and Sokolowski (1988) and Casares and Carracedo (1987) successfully selected strains with significantly different mean pupation heights in food vials (upper dry vs lower wet areas) within 15 generations. Bauer and Sokolowski (1988) also found a significant maternal effect on pupation height, suggesting that high-pupating mothers tend to have high-pupating progeny. In general, they discovered that the main inheritance pattern for the trait was polygenic and additive. Their subsequent study (Sokolowski and Bauer 1989) indicated that factors on the second and third chromosomes contribute additively to variation in the pupation height trait. Here, we are interested in variation in sexual dimorphism of pupation site preference rather than the trait per se. Bauer and Sokolowski (1988) and Casares and Carracedo (1987) documented interesting observations of sexual dimorphism in pupation site preference: male larvae of both *Drosophila simulans* and *D. melanogaster* move further and prefer drier pupation sites than female larvae.

In *Drosophila*, variation in sexual dimorphism is expected to be influenced in part by variation in sex-specific gene expression, which is primarily triggered by the somatic sex determination cascade and dosage compensation systems (Straub and Becker 2007; Verhulst *et al*. 2010; Gempe and Beye 2011; Salz 2011). Because these systems take effect very early in embryogenesis, the male and female states can be considered distinct cellular environments present throughout subsequent development. Thus, nucleotide variation, particularly in cis-acting regulatory elements, may result in differential expression of male- and female-specific gene products throughout development, which is not limited to genes required for the development of existing adult sexual dimorphism. Thus, these variants are putatively available to respond to sex-specific selection for developmental traits.

To test for such variation in sexual dimorphism, we examined the development time and the pupation site preference in 21 *D. melanogaster* DGRP lines (Mackay *et al*. 2012) in 4 replicate experiments conducted under nearly identical environmental conditions. We performed 2 more replicates to confirm the consistency of sexual dimorphism of pupation site preference in 6 lines. Both traits exhibited abundant genetic variation in sexual dimorphism. Here, we present data on the variation in sexual dimorphism among lines and discuss their implications for the evolvability of sexual dimorphism during development.

## Materials and methods

### Fly media

Fly media were prepared with 14 g agar, 70 g brewer's yeast, and 70 g glucose per 1 L water (water volume in Trial VI was decreased to 0.8 L for the same amount of ingredients for controlling the cotton roll moisture level). Additionally, we added a small amount of acid mix to lower the pH and inhibit fungal growth (1 L water to 0.35 mL phosphoric acid and 3.5 mL propionic acid). Reusable plastic vials were autoclaved and thoroughly dried before food dispensing. The agar and yeast were added to boiling water and boiled for 10 min until completely dissolved (for 5 L of food; time varied according to batch volume); once the temperature dropped to 60–70°C, the glucose and acid mixture was added. Each vial was dispensed with 7 mL of food using a dispenser. Food vials were chilled overnight at 4°C and brought up to room temperature before use.

### *Drosophila* strains and culture

We arbitrarily selected and cultured 21 DGRP lines (Mackay *et al*. 2012), which are highly inbred *D. melanogaster* lines derived from a natural population in North Carolina. These lines were cultured in an incubation chamber under uniform environmental settings: 25°C, 30–50% relative humidity, and 12/12 h light/dark cycle. For each line, we set up 10 vials with 5 pairs in each vial to propagate. Then, to produce the experimental flies, for each line, we collected mated female offspring from the propagation vials and allocated 20 females into each of 8 new vials; lines with an insufficient number of mated females were established with fewer vials. In the initial trial, these females laid eggs for 40 h and were removed from the vials. Given that a longer egg-laying time window may result in a relatively high population density, which may interfere with pupation site preference, we reduced the egg-laying time to 24–32 h in the following 5 trials.

One day after removing females from the vials, we inserted a dry cylindrical cotton roll (0.95 × 3.8 cm, 100% cotton, unbraided, Richmond Dental Co. Cat. # 216206) to a depth of ∼15 mm in the center of the food (vial size 2.5 × 9.5 cm). Within a few days, the late third instar larva entered the wandering stage and pupated on the cotton roll or the side of the vial and food surface. Once observing that all larvae in the majority of the vials had pupated, we transferred the cotton rolls to new food vials (Supplementary Fig. 1a). We sexed and counted emerged flies in both the original vial and the vial with the cotton roll 1–3 times per day in most of the trials.

Four trials were conducted with overlapping sets of lines and varying in the conditions described previously. The vial positions in trays and the incubator were not randomized in the initial trial. Randomizing strain and vial placement is expected to control for heterogeneous environmental influences, such as variation in light intensity that may affect pupation site preferences (Manning and Markow 1981). Following Trial I, we revised the protocol by randomly assigning vials to all positions in the incubator. Prior to initiating Trial IV, we reordered DGRP strains (from the Bloomington *Drosophila* Stock Center) and propagated the strains for 2 generations using our food recipe. Given the generally consistent mean differences in pupation site preference between sexes in this sample of lines, we chose 2 lines that showed more males than females pupating on cotton (male cotton, 304 and 362), 2 lines that showed the opposite pattern (427 and 732), and 2 lines with a nonsignificant mean difference between sexes (335 and 517, *P* > 0.05, *t*-test), across the first 4 trials. In Trials V and VI, these 6 lines were evaluated for the repeatability of their cross-sex mean difference patterns in pupation site preference. The trial setup details are summarized in Supplementary Fig. 1b. The data are deposited in Dryad (https://datadryad.org/stash/share/EA4OYK6w-ajNz232YvXT8vfwaS5JqRgywPt2R_6Xiww).

To determine the correlation of sexual dimorphism patterns between the developmental traits and adult morphological traits, we dissected ∼300 pairs of flies from the 6 lines reared in Trials V and VI (adult flies from Trial III preserved in 95% ethanol + 5% glycerin) and measured the wing length, wing width, and abdominal sternite bristle number (Supplementary Fig. 2). We performed *t*-tests on the measured traits between the flies pupated on cotton and not on cotton (Supplementary Table 1). We built linear mixed models for the response variable time and cotton ratio with the independent variables wing length, width, and bristle numbers to males and females in the 3 groups of lines. We performed ANOVA (package stats, R Core Team 2020) on the fitted model. We plotted a correlation heatmap for the sexual dimorphism of these traits in Supplementary Fig. 3.

### Statistical analysis

The data analysis was carried out using R (R Core Team 2020; R Studio Team 2020). The R code is deposited in Dryad (https://datadryad.org/stash/share/EA4OYK6w-ajNz232YvXT8vfwaS5JqRgywPt2R_6Xiww).

### Linear mixed model specifications for the 2 traits

We used linear mixed models to estimate the variance of sexual dimorphism in development time and pupation site preference among the 21 DGRP lines in Trials I to IV, via restricted maximum likelihood (REML) and BOBYQA optimization (Powell 2009) using the function *lmer* (package lme4; Bates, Mächler *et al*. 2015). We fitted the response as the mean difference between the sexes (the female was set as the reference group) to the fixed effects and the random effects. For the development time, the response was the difference in hours between estimated time of egg-laying and estimated time of eclosion. Estimated time of egg-laying was the halfway point of the egg-laying time window. Estimated time of eclosion for the first collection was the collection time. Estimated time of eclosion of later collections was midway point between time of last collection and current collection time. For the pupation site preference, the response was the difference in cotton ratio: percent of flies pupated on the cotton roll.

The fixed effects include “density level,” #collected in a vial, categorized as “low” if below the median 97 (vials of Trials I–IV) or “high” if above; “trial condition,” Trial I vs Trials II–IV, to test the effect of different trial setups; and average “#vials set up per line” of Trials I–IV, categorized as “low” if below the average of all lines (7.06) and “normal” if above. The random effects include “trial” and “line” as accounting for the group effect (random intercept) from different trials and lines. We tested the interaction effects between the fixed effects and between the fixed and random effects (random slope). See Table 1 for the summary of the models.

**Table 1.** Summary table of models for pupation site preference and development time for the 21 lines in Trials I to IV.

Pupation site preference (%pupated on cotton)					95% CI of coefficient	
Fixed effects	Estimate of coefficient	SE of coefficient	*P*		Lower	Upper
Intercept	0.10	0.04	0.01		0.03	0.17
Density in level (D)	−0.08	0.04	**0.03**		−0.15	−0.01
Trial condition (T)	−0.04	0.04	0.25		−0.11	0.03
#Vial per line in level (V)	−0.10	0.05	**0.03**		−0.19	−0.01
D * T	−0.01	0.04	0.74		−0.10	0.07
D * V	0.06	0.05	0.21		−0.03	0.15
T * V	0.06	0.04	0.19		−0.03	0.14
D * T * V	0.01	0.05	0.87		−0.09	0.11
Random effects	Variance	sd	ICC	*Δdf*	*Δχ^2^*	*P*
Line intercept	0.002	0.045	0.18	1	59.11	<0.0001
Trial intercept	0.0001	0.010	0.011	1	0.19	0.66
Residuals	0.009	0.096				
Development time (h)					95% CI of coefficient	
Fixed effects	Estimate of coefficient	SE of coefficient	*P*		Lower	Upper
Intercept	3.50	1.71	0.05		0.14	6.86
Density in level (D)	1.45	1.78	0.42		−2.04	4.94
Trial condition (T)	0.96	1.63	0.56		−2.24	4.16
#Vial per line in level (V)	0.10	2.14	0.96		−4.09	4.29
D * T	−1.05	1.93	0.59		−4.83	2.73
D * V	−1.47	2.27	0.52		−5.92	2.98
T * V	−1.38	1.98	0.49		−5.26	2.50
D * T * V	1.85	2.39	0.44		−2.84	6.53
Random effects	Variance	sd	ICC	*Δdf*	*Δχ^2^*	*P*
Line intercept	4.98	2.23	0.20	3	112.00	<0.0001
Density in level|line	2.60	1.61	0.12	2	6.79	0.03
Trial intercept	0.25	0.50	0.01	1	0.24	0.62
Residuals	19.63	4.43				

Bold: *P* < 0.05. Trial condition is categorized as Trials I vs Trials II–IV, to evaluate the potential environmental effects of the different trial setups (see Supplementary Fig. 1b).

### Estimating the density effect

We estimated the density effect quantitatively for development time. We used only Trial I data and the average time of the sexes within the mean ± 1.5 * standard deviations (sd) (88% of the data).

For pupation site preference, we tested the density level effect on male and female separately. We fitted a generalized linear mixed model to Trials I–IV, with logit-link to the response of the male or the female cotton ratio, which follows a binomial distribution.

### Testing the consistency of pupation site preference in the 6 lines

To test if the mean difference in pupation site preference between sexes was generally consistent between Trials I–IV and Trials V and VI, we pooled the data from the 6 selected lines (see above) and fitted a linear mixed model. The fixed effects included “density level”; “trial sets,” Trials I–IV vs Trials V and VI; and the patterns showed in Trials I–IV, “male cotton” for lines 304 and 362, “female cotton” for lines 427 and 732, and the reference level nonsignificant mean difference between sexes for lines 335 and 517. See Table 2 for a summary of the model.

**Table 2.** Summary table of models for pupation site preference for the 6 selected lines in all the trials (Trials I–VI).

Pupation site preference (%pupated on cotton)				95% CI of coefficient	
Fixed effects	Estimate of coefficient	SE of coefficient	*P*	Lower	Upper
Intercept	−0.02	0.02	0.33	−0.05	0.02
Density in level (D)	0.02	0.03	0.43	−0.03	0.08
Trial sets (T)	0.06	0.03	0.09	−0.01	0.13
Female cotton, lines 427 and 732 (FC)	−0.06	0.03	**0.02**	−0.12	−0.01
Male cotton, lines 304 and 362 (MC)	0.13	0.03	**<0.001**	0.07	0.19
D * T	−0.05	0.05	0.33	−0.15	0.05
D * FC	−0.03	0.04	0.44	−0.11	0.05
D * MC	−0.06	0.04	0.17	−0.14	0.02
T * FC	0.05	0.05	0.33	−0.05	0.14
T * MC	0.03	0.05	0.57	−0.07	0.13
D * T * FC	−0.01	0.07	0.93	−0.14	0.13
D * T * MC	−0.14	0.07	0.06	−0.28	0.01
Pupation site preference (%pupated on cotton)				95% CI of coefficient	
Fixed effects	Estimate of coefficient	SE of coefficient	*P*	Lower	Upper
Intercept	−0.02	0.02	0.33	−0.05	0.02
Density in level (D)	0.02	0.03	0.43	−0.03	0.08
Trial sets (T)	0.06	0.03	0.09	−0.01	0.13
Female cotton, lines 427 and 732 (FC)	−0.06	0.03	**0.02**	−0.12	−0.01
Male cotton, lines 304 and 362 (MC)	0.13	0.03	**0.000**	0.07	0.19
D * T	−0.05	0.05	0.33	−0.15	0.05
D * FC	−0.03	0.04	0.44	−0.11	0.05
D * MC	−0.06	0.04	0.17	−0.14	0.02
T * FC	0.05	0.05	0.33	−0.05	0.14
T * MC	0.03	0.05	0.57	−0.07	0.13
D * T * FC	−0.01	0.07	0.93	−0.14	0.13
D * T * MC	−0.14	0.07	0.06	−0.28	0.01

Bold values indicate statistically significant *P* - values (*P* < 0.05).

Trial condition is categorized as Trials I–IV vs Trials V–VI, to test the consistency of the sexual dimorphism pattern in the selected lines (see Materials and methods) between the trials. The female cotton (FC) and male cotton (MC) are levels of a categorical variable, and the reference level is the “expected to show no sexual dimorphism” of lines 335 and 517.

### Estimating the magnitude of variation in sexual dimorphism in lines

**Fig. 1.**
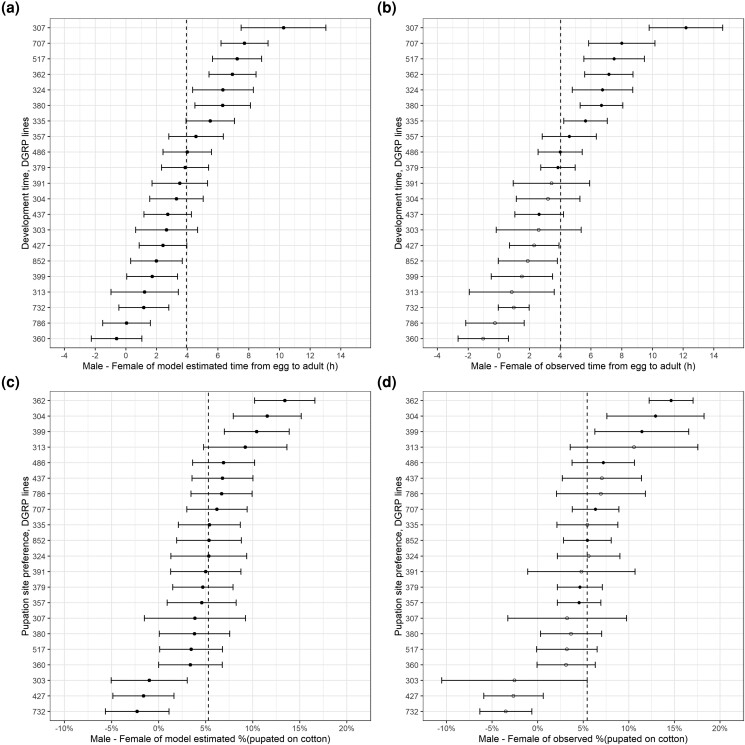
The model-estimated and observed differences between the sexes in development time (a and b) and pupation site preference (c and d) in the 21 DGRP lines. The filled circles in b) and d) represent statistical significance after the Bonferroni correction for multiple within-line *t*-tests (different from sexual monomorphism). The vertical dashed line denotes the average of the 21 lines of each panel. The high consistency (Pearson's *r*) between the left and right panels suggests that the model provides an accurate estimate of the observed sexual dimorphism.

We obtained the mean and sd of the random intercept for each line using the function *ranef* (package lme4; Bates, Mächler *et al*. 2015). We then plotted the (mean ± 1.96 * sd) for each line in Fig. 1a and c, as the model-estimated mean difference between sexes of the development time and cotton ratio (function *ggplot*, package ggplot2; Hadley 2016). For Fig. 1b and d, we performed *t*-tests to compare males and females within each individual line and then aligned the between-sex differences with a) and c). We then compared the model estimated with the observed sexual dimorphism using Pearson's correlation *r* (function *cor.test*, package stats; R Core Team 2020).

To estimate the effect size of the sexual dimorphism among lines, we derived the intraclass correlation coefficient (Snijders and Bosker 2013; ICC, Lorah 2018) as the “between-group variance (e.g. variance of “Line intercept” in the “Random effects”; see Table 1)/(between-group variance + the model residual).”

## Results and discussion

We scored ∼71,000 adult flies (49.6% male) from 623 vials over the 6 trials. A sample of 17–21 DGRP inbred lines was used in the first 4 trials, and, from those, 6 lines were selected for further testing in the final 2 trials. Each trial sampled an average of 819 adults per line and 114 adults per vial (sd = 74.2). Males, on average, took 286.3 h (sd = 26.7) from egg to adult, while females took 282.5 h (sd = 27.1), across all lines and trials.

Flies that pupated on cotton emerged significantly later than flies that pupated on the side of the vial or top of food medium (difference in males 8.6 h, *P* < 0.001; in females 7.8 h, *P* < 0.001). In general, larvae that chose the vial side or food surface started pupating earlier than the larvae that chose the cotton. This indicates that a preference for pupating on cotton is related to an increased amount of time spent in the developing, feeding, or wandering stages or possibly a later average start time to development.

Approximately 24% of flies pupated on cotton. Males pupated on cotton at a higher rate (by about 1%) than females, but the difference was not statistically significant [*P* = 0.28, 95% CI (−0.01, 0.04), *t*-test]. However, a significant interaction between sex and vial density was observed, suggesting that environmental factors may obscure the overall sexual dimorphism (Table 1; pupation site preference, density in level *P* = 0.01). Additionally, by fitting the linear mixed models for the trait, we observed considerable genetic variation in sexual dimorphism among the 21 DGRP lines.

### Sexual dimorphism in development time

We observed a substantial variance in the sexual dimorphism among lines [Table 1; development time, line intercept *P* < 0.0001, likelihood ratio test (LRT)]. We aligned the model estimate with the observed mean differences between sexes (*t*-test) in Fig. 1a and b. This plot can help visualize the magnitude of sexual dimorphism variation among lines. In most lines (19/21), males required a longer period of development time than females. The most extreme line, 307, showed an additional 6 h of sexual dimorphism (males emerged 10 h later than females compared with the average of 4 h). Lines 786 and 360, on the other hand, showed the opposite direction of sexual dimorphism: females emerged about 1 or <1 h later than males, on average, in these lines (Fig. 1b).

The fixed effects density, trial condition, and average vial per line (see Materials and methods for details) were not significant nor were their interactions (Table 1; *P* > 0.05). This suggests that the difference in vial density does not have a significant effect on the sexual dimorphism in development time. The different setups in the initial trial and the following trials had no significant effect nor the uneven number of vials setup for each line. There was not enough variance to support the random slope structure of these fixed effects on the random effects “trial” and “line” (function *rePCA*, package lme4; Bates, 2015). In summary, these effects due to uncontrolled environmental conditions within or across trials appear to have no significant impact on the sexual dimorphism in development time.

To test the density effect quantitatively, we fitted the average development time of the males and females to the Trial I data. We found that the density effect was highly significant and had a positive correlation with development time (estimate = 0.051, *P* = 0.003). This indicates that for every increase of 100 larvae in the vial *density* (100 additional adults observed in that vial), the development time for both sexes was lengthened by 5.1 h. Larvae developing in high-density vials spent more time on average foraging, wandering, and/or in pupal development prior to eclosion.

In summary, we found that in the majority of the 21 DGRP lines, males required significantly more development time than females. We found a significant positive correlation between larval density and development time (5.1 h per 100 individuals). Males and females responded similarly in this trait to increasing larval crowdedness. The model-estimated sexual dimorphism was consistent with the observed sexual dimorphism in the lines. We discovered substantial variation in sexual dimorphism in development time among the DGRP lines. There would thus appear to be abundant genetic variation in sexual dimorphism for this trait in the natural *D. melanogaster* population from which the DGRP lines were sampled.

### Sexual dimorphism in pupation site preference

We discovered substantial phenotypic variation in pupation site preference between sexes among lines (Table 1; pupation site preference, line intercept *P* < 0.0001, LRT). The mean differences between the sexes were plotted in Fig. 1c and d (the density effect was offset in d). Generally, in 18/21 lines, more males than females pupated on cotton. However, lines 732,427, and 303 exhibited a pattern in the opposite direction, with more females than males pupating on cotton. Overall, these results indicate that sexual dimorphism varied significantly among lines.

The fixed effects in the development time model were also tested for pupation site preference. The density had a significant effect on the sexual dimorphism of cotton ratio (estimate = −0.08, *P* = 0.01). The ad hoc analysis by fitting generalized linear mixed models for males and females separately showed a negative and significant density effect on males (estimate = −0.32, *P* < 0.0001) and a relatively smaller density effect on females (−0.08, *P* = 0.02). These together suggest that males exhibited this density-dependent pattern more strongly than females. Male larvae in a less crowded vial responded similarly to density as female larvae, whereas in an overcrowded vial, the responses were significantly different. As density increased, males experienced a higher negative influence of density and thus showed a lower preference for pupating on cotton than females. As discussed above, we only observed a nonsignificant 1% difference in cotton ratio between the sexes when density was taken into account (*P* = 0.28, *t*-test). Therefore, environmental factors such as vial density may frequently obscure the observed sexual dimorphism in this trait.

Besides the density effect, the imbalance in the average number of vials set up between lines also significantly affected the sexual dimorphism (estimate = −0.10, *P* = 0.01). Due to the variation in line fecundity, some lines were unable to produce enough females for setting up 8 vials. The estimate of coefficient suggests that the lines with more vials set up (>mean 6.34, 13 lines in total) showed reduced sexual dimorphism in the “male minus female” direction, compared with the lines with less vials set up (8 lines). In the future experiments, more propagation vials should be set up in order to ensure roughly equal sample sizes of lines.

From the first 4 trials, we found lines 304 and 362 showed “male on cotton and female on vial” pattern, lines 427 and 732 showed the opposite pattern, and lines 335 and 517 showed a nonsignificant mean difference between sexes (*P* > 0.05, *t*-test). In Trials V and VI, we evaluated these 6 lines for the repeatability of their cross-sex mean difference patterns in pupation site preference. We found that the “Male cotton, lines 304 and 362” vs the reference level (nonsignificant, lines 335 and 517) and the “female cotton, lines 427 and 732” vs the reference level were both significant (estimate = 0.13, −0.06, *P* = <0.001, 0.02, respectively; Table 2). The fixed effect “trial sets” was categorized as “Trials I–IV” and “Trials V and VI” and was not significant nor was the interaction with the group effects. This suggests that the pupation site preference patterns of the group of lines did not change significantly in the last 2 trials compared with the first 4. The patterns were thus generally consistent across trials.

In summary, we discovered a significant density effect on sexual dimorphism, and males were more sensitive to high vial density than females, making cotton a less utilized pupation substrate for males in crowded vials. After controlling for the density effect, we found that in 18/21 of the lines in our experiment, more males than females pupated on the cotton. The 6 selection lines from the first 4 trials showed generally consistent pupation site preference patterns when evaluated in the last 2 trials. In conclusion, the wide distribution of sexual dimorphism in inbred lines suggests considerable genetic variation in sex-specific pupation site preference. These 21 DGRP lines clearly contain abundant genetic variation for sexual dimorphism in the trait, despite apparent environmental factors such as vial density that may frequently obscure it.

### ICCs

The ICC (the variance of random effect at interest to the model residual; see Materials and methods for details) for the development time trait was 0.20, which represents a moderate effect size of the sexual dimorphism among lines (see Table 1) and is comparable to the sexual size dimorphism 0.21 found in David *et al*. (2003) from 30 *D. melanogaster* lines. For the pupation site trait, the ICC was 0.18, suggesting that the variance in sexual dimorphism among lines for the pupation site preference trait was slightly lower than the development time trait but still significantly different from 0 (LRT, see above).

### Correlations between larval and adult sexual dimorphism

Correlations in sexual dimorphism between distinct traits could be due to common regulatory mechanisms, such as the somatic sex determination system acting similarly on target genes involved in development of the traits. To determine whether the degree and direction of sexual dimorphism are correlated between the larval traits studied here and between these traits and sexually dimorphic adult morphological traits, we dissected ∼300 pairs of flies from the 6 strains tested in Trials V and VI, measured wing size, and counted bristles on the abdominal sternites (Supplementary Fig. 2). The flies examined were chosen randomly from those that emerged in Trial III. Previously characterized patterns of sexual dimorphism were seen in the adult traits: females in all 6 strains had larger wings (see e.g. Houle *et al*. 2003) and more abdominal sternite bristles than males (see e.g. Mackay and Fry 1996). We performed ANOVA on the response variable time and cotton ratio to the wing width, length, and number of bristles as the independent variable, in groups of males and females and in the 3 groups of lines. The lines showed distinct sexual dimorphism in pupation site preference: male cotton (304, 362), female cotton (427, 732), and no significant difference in cotton preference (335, 517). We observed weak correlations between the sexual dimorphism in cotton ratio and in wing width (*P* = 0.08) and in number of bristles (*P* = 0.04). This indicates that there was not a strong pattern of correlation between the larval and the adult traits we measured.

If there were strong correlations between sexual dimorphism in the larval and adult traits, we would expect the contrast between the strongest “male cotton” lines (304 and 362) and the strongest “female cotton” lines (427 and 732) to show parallel patterns in the bristle and wing traits (i.e. 304 and 362 strong sexual dimorphism in 1 direction and 427 and 732 showing strong sexual dimorphism in the opposite direction). This pattern was not seen for any of the morphological traits (Supplementary Fig. 3; no strong positive correlation between the sexual dimorphism of cotton preference vs the sexual dimorphism of wing length/width or the bristle numbers). Similarly, no correlation between sexually dimorphic patterns of the 2 larval traits was observed (*P* = 0.26, ANOVA). These results are consistent with variation in sexual dimorphism in distinct traits being genetically independent.

It is thus reasonable to infer that the genetic variation underlying variability in sexual dimorphism in *D. melanogaster* resides in genes downstream of the sex determination pathway rather than in major regulatory genes (e.g. *dsx*, *tra*, *fru*). This is consistent with a meta-analysis study in mice by Zajitschek *et al*. (2020), which reached a similar conclusion. Given that dosage compensation is associated with a high proportion of sex-biased gene expression in *D. melanogaster* (Chang *et al*. 2011), it would be interesting to determine whether a substantial proportion of genetic variation underlying sexual dimorphism is X-linked. Variation in the dosage compensation of specific genes between strains may provide genetic variation for the evolution of sexual dimorphism for specific traits.

Our finding of genetic variation in sexual dimorphism suggests that the traits investigated have presumably not undergone strong selection for sex differences in the recent evolution of this lineage. Nevertheless, the effect sizes of the sexual dimorphism for both traits are comparable with those for sexual size dimorphism (ICC, see above; David *et al*. (2003)), in which variation has been found to be considerable in different insect species (Stillwell *et al*. 2010). Further investigation of variation of sexual dimorphism in these traits in additional populations and in related species could shed light on the history of selection on this variation. For example, if populations or species differ in the amount of standing variation in sexual dimorphism in a particular trait, this would suggest different histories of selection on sexual dimorphism in the trait.

Overall, we observed considerable variation in sexual dimorphism among DGRP lines in both development time and pupation site preference. These patterns of sexual dimorphism were fairly consistent across 6 trials. These findings suggest abundant genetic variation in sexual dimorphism for *D. melanogaster* in natural populations and that a lack of selection likely explains the lack of substantial, fixed sexual dimorphism in these traits in this species. Environmental factors likely exert large effects in masking genetic variation in sexual dimorphism. Elucidating the functional basis of variation in sexual dimorphism in natural populations will likely provide valuable insights on the evolvability of sex differences and the structures of developmental pathways underlying sex differences in animals.

## Supplementary Material

jkae010_Supplementary_Data

## Data Availability

All data from this study are deposited in Dryad (True *et al.*, 2023; https://datadryad.org/stash/share/EA4OYK6w-ajNz232YvXT8vfwaS5JqRgywPt2R_6Xiww). Supplemental material available at G3 online.
