# Supplementary material for: Genetic variation for sexual dimorphism in developmental traits in Drosophila melanogaster
Source: G3 (Bethesda). 2024 Mar 1;14(4):jkae010. doi: 10.1093/g3journal/jkae010 (PMC10989870; doi:10.1093/g3journal/jkae010)

Figure S1

(A) Diagram of the *Drosophila* culture vial (plastic), the food medium on the bottom, and the cotton roll inserted into the center of the food medium for larva to pupate. (B) Trials setup details.

A.

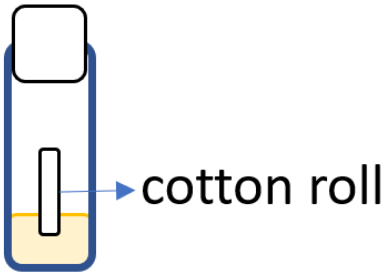

B.

Trial	# lines in trial	Vial position randomization?	Oviposition duration (h)	Average density (per vial)	Note:
I	21	No	40	175	-
II	20	Yes	28	107	-
III	17	Yes	24	74	-
IV	18	Yes	24	80	Reordered DGRP lines
V	6	Yes	30	130	Selected lines only
VI	5	Yes	32	112	Selected lines only

Table S1

t-tests between flies pupated on vial and on cotton for measured adult traits. (M=mean, SD=standard deviation)

Traits		Vial (n=313)	Cotton (n=333)	*t*	*p*
Wing Length	M	1.401	1.405	-0.63	0.531
	SD	0.097	0.099		
Wing Width	M	0.926	0.930	-0.76	0.446
	SD	0.068	0.072		
Sternite 2	M	12.00	11.68	1.68	0.093
	SD	2.54	2.16		
Sternite 3	M	16.97	17.27	-1.26	0.208
	SD	2.92	3.00		
Sternite 4	M	16.93	16.89	0.18	0.859
	SD	3.13	2.97		
Sternite 5	M	16.92	16.61	1.20	0.229
	SD	3.16	3.31		

Figure S2

(A) Wing length was measured as distance between the intersection of anterior crossvein with L4 to the intersection of L3 with the wing margin, and the wing width as the distance between the intersection of the posterior crossvein with L5 to the intersection between L2 and the wing margin. (B) The number of bristles were counted in abdominal sternites 2, 3, 4 and 5 in both males and females.

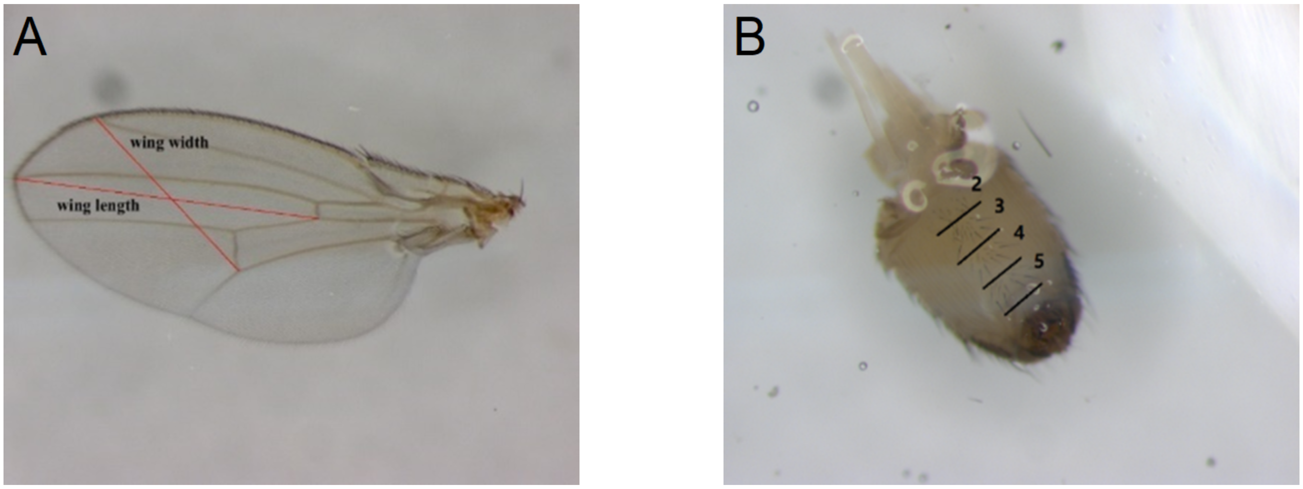

Figure S3

Correlation heatmap of sexual dimorphism between the developmental traits (development time and cotton preference) and adult morphological traits (wing size and bristle number on sternites).

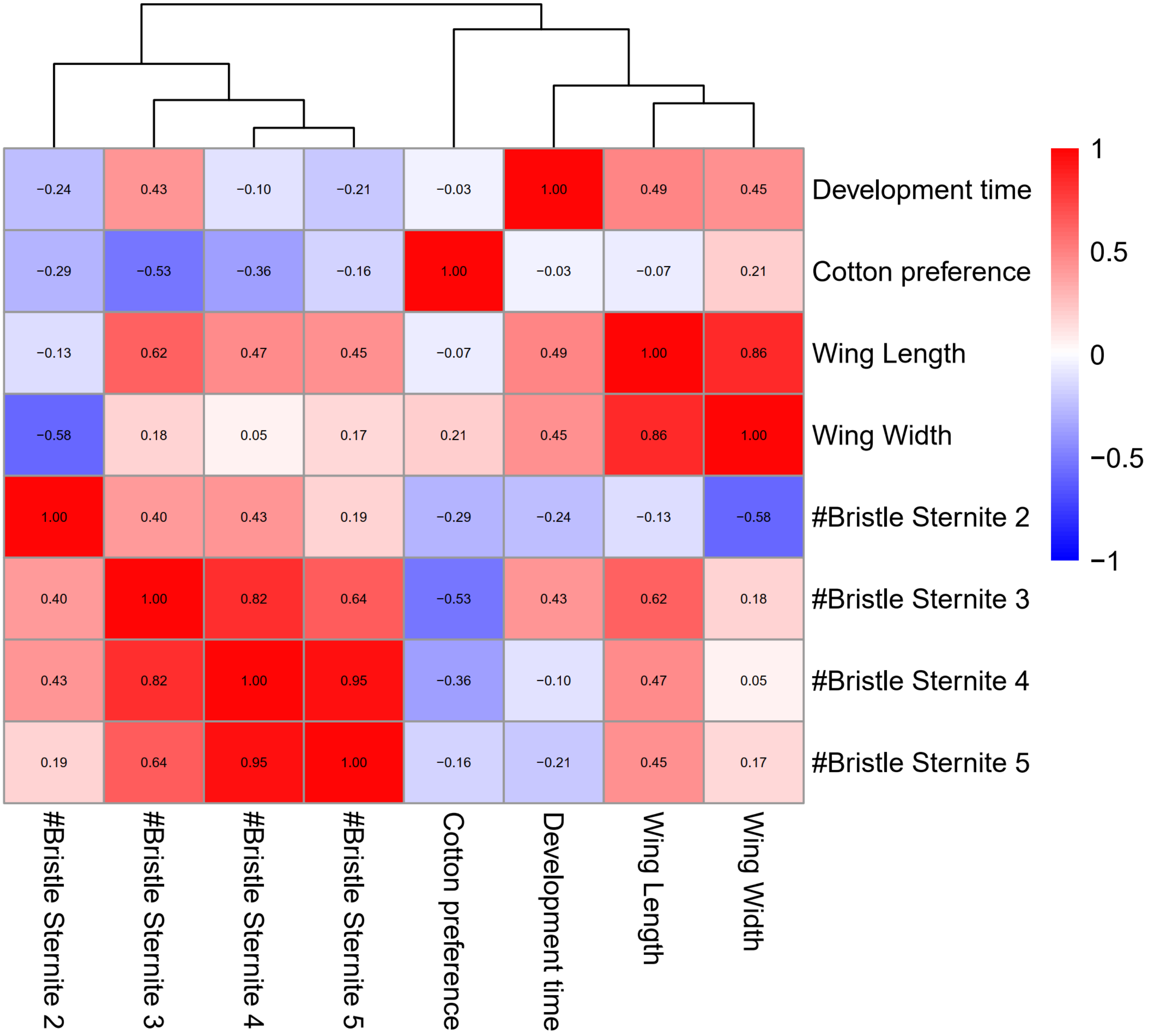

Supplement: jkae010_Supplementary_Data [file jkae010_supplementary_data.pdf]
